# Dye-Decolorizing Peroxidases Maintain High Stability and Turnover on Kraft Lignin and Lignocellulose Substrates

**DOI:** 10.1021/acsomega.4c05043

**Published:** 2024-10-31

**Authors:** Silja Välimets, Lorenz Schwaiger, Alexandra Bennett, Daniel Maresch, Roland Ludwig, Stephan Hann, Dolores Linde, Francisco Javier Ruiz-Dueñas, Clemens Peterbauer

**Affiliations:** † Department of Food Science and Technology, Institute of Food Technology, BOKU University, Muthgasse 11, 1190 Vienna, Austria; ‡ Doctoral Programme BioToP – Biomolecular Technology of Proteins, BOKU University, Muthgasse 18, 1190 Vienna, Austria; § Department of Chemistry, Institute of Analytical Chemistry, BOKU University, Muthgasse 18, 1190 Vienna, Austria; ∥ Core Facility Mass-spectrometry, BOKU University, Muthgasse 11, 1190 Vienna, Austria; ⊥ Centro de Investigaciones Biológicas Margarita Salas (CIB), Consejo Superior de Investigaciones Científicas (CSIC), Ramiro de Maeztu 9, 28040 Madrid, Spain

## Abstract

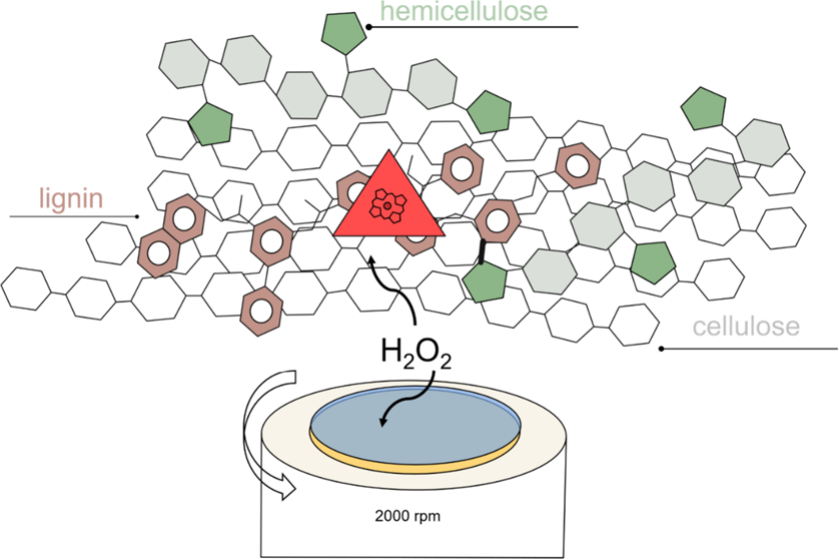

Fungal enzyme systems for the degradation of plant cell wall lignin, consisting of, among others, laccases and lignin-active peroxidases, are well characterized. Additionally, fungi and bacteria contain dye-decolorizing peroxidases (DyP), which are also capable of oxidizing and modifying lignin constituents. Studying DyP activity on lignocellulose poses challenges due to the heterogeneity of the substrate and the lack of continuous kinetic methods. In this study, we report the kinetic parameters of bacterial DyP from *Amycolatopsis* 75iv2 and fungal DyP from *Auricularia auricula-judae* on insoluble plant materials and kraft lignin by monitoring the depletion of the cosubstrate of the peroxidases with a H_2_O_2_ sensor. In the reactions with spruce, both enzymes showed similar kinetics. On kraft lignin, the catalytic rate of bacterial DyP reached 30 ± 2 s^–1^, whereas fungal DyP was nearly 3 times more active (81 ± 7 s^–1^). Importantly, the real-time measurement of H_2_O_2_ allowed the assessment of continuous activity for both enzymes, revealing a previously unreported exceptionally high stability under turnover conditions. Bacterial DyP performed 24,000 turnovers of H_2_O_2_, whereas the fungal DyP achieved 94,000 H_2_O_2_ turnovers in 1 h with a remaining activity of 40 and 80%, respectively. Using mass spectrometry, the depletion of the cosubstrate H_2_O_2_ was shown to correlate with product formation, validating the amperometric method.

## 1 Introduction

The plant cell wall is composed of a complex architecture of recalcitrant polymers that provide structural strength and protection against microbial invasions. While cellulose together with hemicelluloses constitutes the majority of biopolymers, lignin presents the most challenging and heterogeneous properties.^1^ Originating from the Latin word “lignum” meaning wood, lignin is a highly cross-linked aromatic polymer formed through the oxidative coupling of monolignols such as p-coumaryl alcohol, coniferyl alcohol, and sinapyl alcohol together with other more recently discovered monomers.^1,2^ The common linkages between monolignols are condensed (β-5, β-β, β-1, 5-5) or ether (β-*O*-4, 4-*O*-5) bonds, of which the β-*O*-4 bond accounts for around 60% of the total linkages.^1,3^ Despite the structural rigidity of lignin, it is still susceptible to microbial degradation. Fungal enzyme systems, which are capable of lignin depolymerization via radical-mediated strategies through the oxidative action of heme- and multicopper-dependent enzymes such as lignin peroxidases, versatile peroxidase, manganese peroxidase, dye-decolorizing peroxidase (DyP), and laccases, have been well characterized.^4,5^

Besides fungi, as the main degraders of lignocellulose, the role of bacteria is often overlooked and underexplored. Numerous bacteria have demonstrated the ability to utilize lignocellulosic material as their sole carbon source^6−8^ leading to an upregulation of oxidoreductase genes.^9,10^ The first evidence of bacterial lignin degradation surfaced through the examination of *Streptomyces viridosporus* T7A, which unveiled the presence of lignin peroxidase-like activity in the secretome.^11,12^ However, fungal-like heme peroxidases occur rarely in bacterial genomes; thus, the oxidative capability for degrading lignocellulose in bacteria predominantly relies on dye-decolorizing peroxidases, which are able to oxidize the phenolic moiety of lignin.^13,14^

**Figure 1:**
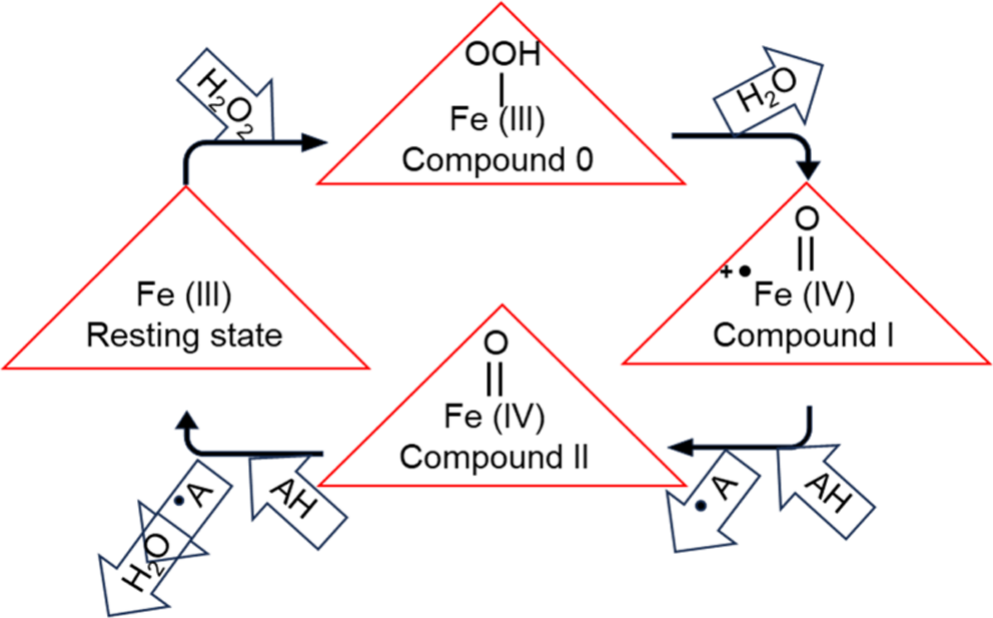
Catalytic cycle of dye-decolorizing peroxidase. The cycle is initiated by the deprotonation of hydrogen peroxide, followed by the formation of short-lived Compound 0. Next, Compound I is formed by the heterolytic cleavage of hydrogen peroxide from Compound 0. Upon the oxidation of one substrate (AH), Compound I is reduced to Compound II. Finally, another oxidation of the substrate molecule returns the enzyme from the Compound II state to the resting state.

The catalytic cycle of dye-decolorizing peroxidases is initiated by the deprotonation of H_2_O_2_ followed by the formation of a short-lived ferric hydroperoxide complex or Compound 0. The Compound I intermediate is formed from the heterolytic cleavage of hydrogen peroxide from Compound 0. Compound I is reduced to Compound II by the oxidation of one substrate molecule, followed by the oxidation of a second substrate molecule, ultimately returning the enzyme to its ferric resting state (Figure 1).^15,16^ Thus, monitoring the conversion of H_2_O_2_ enables kinetic characterization of peroxidases. Conventional methods for detecting hydrogen peroxide often encompass fluorimetry, chemiluminescence, fluorescence, and spectrophotometry.^17−19^ However, these approaches are severely hampered by insoluble materials, such as bulky lignin or lignin-derived soluble compounds. The presence of aromatic moieties in these compounds leads to absorption in the ultraviolet–visible light (UV/vis) region, thereby generating interference background signals for the assays.

Rather than tracking H_2_O_2_ conversion as a measure of enzyme activity, the structural analysis of the target polymer is often carried out using different configurations of mass spectrometry (MS), nuclear magnetic resonance (NMR), Fourier transform infrared spectroscopy (FT-IR), or gel penetration chromatography (GPC).^20,21^ Although these sophisticated methods reveal modifications on polymeric lignocellulose and identify specific products, they demand special expertise and highly advanced technical equipment. Therefore, the information on catalytic activity with lignocellulosic substrates remains limited, largely due to the absence of simple analytical methods.^22−24^

To overcome the complexity of lignocellulose, DyP activity is often studied using model compounds such as 2,2′-azino-bis (3-ethylbenzothiazoline-6-sulfonic acid) (ABTS), 2,6-dimethoxyphenol (DMP), various dyes or dimeric lignin compounds phenolic guaiacylglycerol-β-guaiacyl ether (GGE) and nonphenolic veratrylglycerol-β-guaiacyl ether (VGE).^14,23,25−30^*In vitro* studies of bacterial and fungal DyPs have shown the modification of lignin model compounds via the cleavage of β-*O*-4 bonds or the formation of oxidatively coupled products.^14,23,25−28,31^ However, these model compounds are only representatives of the natural substrates and do not mirror the real conditions. For thorough investigations of pathways for modification, degradation, and valorization of lignocellulose in general and of the lignin fraction in particular by enzymatic processes, it is crucial to develop methods that allow an assessment of enzymatic reactions on natural or near-natural, complex lignocellulosic substrates and that help in optimizing reactions with complex substrates to industrial requirements.

In response to the current challenges posed by the lack of simple analytical methods and the complexity of natural substrates, we screened the activity of bacterial DyP from *Amycolatopsis* 75iv2 and fungal DyP from *Auricularia auricula-judae* on heterogeneous lignocellulosic substrates using a novel H_2_O_2_ sensor.^32^ The tested substrates included chemically untreated corn, sugarcane, wheat straw, mate tea residues, poplar, birch, spruce, and processed kraft lignin. Furthermore, we compared the stability under turnover conditions of the two enzymes, revealing the catalytic differences and high turnover performances. We verified the conversion of H_2_O_2_ by analyzing the reaction products with nontargeted high-resolution mass spectrometry.

## 2 Results and Discussion

### 2.1 Screening Dye-Decolorizing Peroxidase Activity on Lignocellulosic Substrates

A recent development in hydrogen peroxide measurements has introduced a sensor tailored for the determination of lytic polysaccharide monooxygenase (LPMO) activity on heterogeneous insoluble substrates.^32^ Notably, this amperometric H_2_O_2_ sensor exhibits versatility beyond LPMO and carbohydrate substrates, extending its applicability to peroxidases and aromatic polymers.

We selected DyP2 from *Amycolatopsis* 75iv2, which is the most active bacterial DyP currently known^25^ and screened its activity with various lignin and lignocellulosic materials such as kraft lignin, residues from the food industry (mate tea), untreated plant materials (corn cobs, wheat straw, sugarcane), and untreated wood materials (spruce, birch, poplar). The substrates (100 g L^–1^ with a particle size smaller than 125 μm) were resuspended in sodium acetate buffer at pH 4.5 and incubated overnight on an orbital shaker. The high substrate loading was chosen to prevent peroxidase self-inactivation caused by lack of substrate.^33,34^

**Figure 2:**
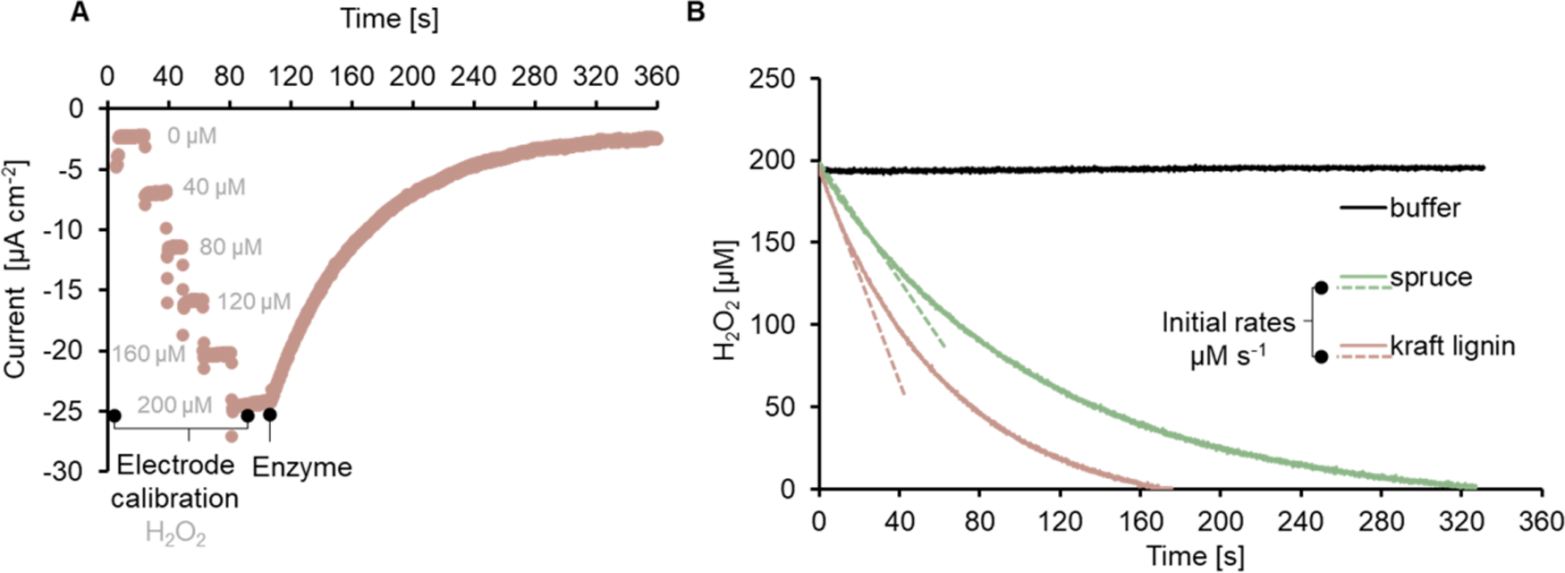
Overview of the H_2_O_2_ sensor for measuring peroxidase activity in real time. (A) Each measurement begins with a stepwise calibration of the rotating disc electrode with H_2_O_2_ in the presence of the substrate. A stable current after each titration step indicates the absence of side reactions between H_2_O_2_ and the substrate, thereby serving as a negative control. Subsequently, the reaction is initiated by the addition of the enzyme, and any rapid change in current is caused by enzyme activity. (B) Time trace measurements of bacterial DyP2 on kraft lignin and spruce as examples. Substrate (100 g L^–1^) is resuspended in 50 mM sodium acetate pH 4.5 buffer, a total of 200 μM H_2_O_2_ is gradually titrated to calibrate the sensor, and the reaction is initiated by the addition of the enzyme. The measured current is converted to H_2_O_2_ concentration by utilizing the electrode calibration function. The initial rates are determined using linear regression from 0 to 40 or 50 s from the reaction start. Buffer instead of substrate solution is used for the negative control.

After overnight preparation of the substrates, each measurement began with the calibration of the Prussian blue-modified gold electrode. While rotating the electrode to ensure a high rate of mass transport, a controlled steady-state concentration of the substrate, and a fast response time, 40 μM H_2_O_2_ is added in a stepwise fashion to a final concentration of 200 μM in the substrate suspension (Figure 2A). The reaction occurring on the sensor to generate the current is H_2_O_2_ + 2H^+^ + 2e^–^ → 2H_2_O and less than 1% of H_2_O_2_ is consumed for the detection reaction.^32^ The reaction is initiated by the addition of the peroxidase, and a rapid increase in negative current indicates H_2_O_2_ conversion by the peroxidase. The measured current is then converted to H_2_O_2_ concentration (μM) plotted against time (s) (Figure 2B) and the initial rates of the peroxidase are determined using linear regression from 40 to 50 s after the initiation of the reaction by enzyme addition.

**Figure 3:**
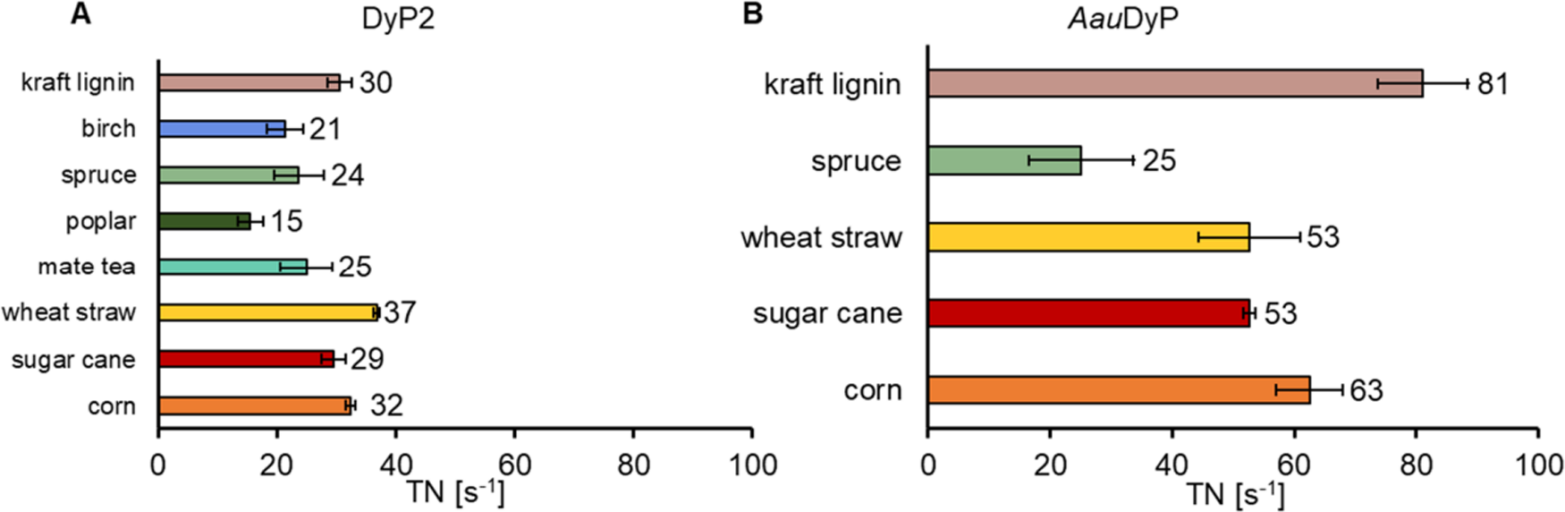
Dye-decolorizing peroxidase activity on lignocellulosic substrates. The turnover number (TN) was obtained by linear regression 40 to 50 s from the beginning of the reaction of H_2_O_2_ sensor time trace measurements. The catalytic rates of (A) bacterial DyP2 and (B) fungal *Aau*DyP on lignocellulosic substrates. The highest catalytic rate of DyP2 was measured on wheat straw, while the lowest was measured on poplar wood. The catalytic rate of fungal *Aau*DyP was nearly 3 times higher compared to the bacterial enzyme on kraft lignin, and nearly 2 times higher on wheat straw, sugarcane, and corn, whereas the catalytic rate on spruce was in a similar range. The data represent the average of three replicates.

Figures 2B and S1 show a rapid decrease in H_2_O_2_ concentration when kraft lignin, spruce, and other lignocellulosic substrates are present, indicating DyP2 reactivity with compounds found in these materials. No changes in the H_2_O_2_ concentration were observed in the control reaction with the buffer alone, indicating that the peroxidase reaction solely occurred in the presence of an electron donor. After normalizing initial rates to the enzyme concentration, the highest catalytic activity of DyP2 was measured with wheat straw (37 ± 1 s^–1^), while the lowest catalytic activity was measured with poplar wood (15 ± 3 s^–1^) (Figure 3A). Despite the identical sample preparation, grasses and wood have distinct cell wall compositions suggesting a potential role of the accessibility of phenolic substrates for activity.^35^ This variance may explain the differences observed in the measured catalytic rates.

The catalytic rate of DyP2 on kraft lignin, the representative substrate of treated lignocellulose, was 30 ± 2 s^–1^, a rate comparable to that observed with untreated plant material. In theory, the lignin extraction process typically results in an increase in aromatic content due to the removal of cellulose and hemicelluloses and thus more accessible substrate for DyP and higher catalytic rates. However, the observed similarity in catalytic rates on kraft lignin and spruce may be explained by several factors, including the destruction of natural bonds, modification of lignin during the extraction process, or the presence of enzyme-inhibiting compounds postextraction,^36−38^ which can be contradictory processes to enhance or limit enzyme activity.

To ensure that DyP2 performed under saturated conditions with complex insoluble substrates, the catalytic rates were determined using different concentrations of kraft lignin and spruce suspensions (Figures S2 and S3). The DyP2 reactions on kraft lignin varying from 1 to 100 g L^–1^ showed no significant difference in catalytic rates, indicating that the enzyme was already running at saturated conditions with 1 g L^–1^ kraft lignin. Similar results were observed with spruce, varying from 1 to 100 g L^–1^. Again, no significant changes in turnover were observed, indicating that the reaction was running under saturated conditions.

To compare the catalytic rates of bacterial *Amycolatopsis* 75iv2 DyP2 on lignocellulose, we selected a fungal DyP from *Auricularia auricula-judae*. Remarkably, the catalytic rates of the fungal *Aau*DyP on kraft lignin were nearly 3 times higher (81 ± 7 s^–1^) compared to those of the bacterial DyP (Figure 3B). Previously, *Aau*DyP activity was demonstrated with both nonphenolic and phenolic lignin model compounds, whereas bacterial DyP2 was only active with phenolic lignin model compounds.^14,25^ This suggests a broader range of substrates for *Aau*DyP in the kraft lignin suspension, potentially contributing to its higher catalytic rates. The catalytic rates of *Aau*DyP on wheat straw, sugarcane, and corn were almost 2 times higher than the catalytic rates of DyP2 on the same substrates. Interestingly, the catalytic rates of *Aau*DyP and DyP2 were identical when spruce was used as the substrate. Thus, the H_2_O_2_ sensor measurements confirm that the used bacterial DyP exhibits comparable activities to fungal DyP as reported earlier on model compounds.^25^ While the comparison of *Aau*DyP and DyP2 was previously studied with lignin dimers,^14^ this work reveals the differences on heterogeneous complex substrates.

During substrate screening shown in Figure 3 with the H_2_O_2_ sensor, the current remained stable throughout electrode calibration, indicating the absence of any side reactions between the substrate solution and H_2_O_2_. However, it should be noted that the sensor is also able to detect an enzyme-independent reaction between H_2_O_2_ and a water-soluble lignin substrate, particularly lignosulfonate. In Figure S4, a change in current is observed during electrode calibration. A rapid increase in negative current was noted upon direct addition of 200 μM H_2_O_2_ to the lignosulfonate suspension in the absence of the enzyme, indicating reactivity with unknown lignosulfonate compounds. Even after 100 s, the current was still not stabilized, contrary to the behavior observed with other studied substrates, which behaved as shown in Figure 2A during electrode calibration. Lignosulfonate, classified as water-soluble technical lignin, can contain various metal ions resulting in the side reaction with H_2_O_2_.^39^ Thus, a stable current measured during electrode calibration signifies no reactivity with H_2_O_2_, rendering it suitable as a negative control.

To confirm that DyP2 was only active with aromatic compounds and not with plant carbohydrates, the activity measurements were performed using chitin, curdlan, mannan, and xylan as substrates. Figure S5 shows no decrease in the concentration of H_2_O_2_, suggesting no occurrence of the peroxidase reaction in the presence of the tested carbohydrates. While background activity was measured with chitin, H_2_O_2_ consumption was only partial and did not return to the baseline of 0 μM compared to kraft lignin, suggesting a potential unknown contamination in the used chitin preparation, which was of technical grade purity. These findings suggest that DyP2 exhibits activity exclusively with plant phenolic compounds.

However, not all bacterial dye-decolorizing peroxidases show activity on lignin. The reaction of DyPA from *Escherichia coli* showed no activity on kraft lignin. This indicates that DyPA cannot utilize aromatic lignin compounds as substrates (Figure S6) suggesting diverse yet unknown physiological roles of dye-decolorizing peroxidases.

### 2.2 Revealing the Kinetic Stability of Dye-Decolorizing Peroxidases on Kraft Lignin and Untreated Spruce

**Figure 4:**
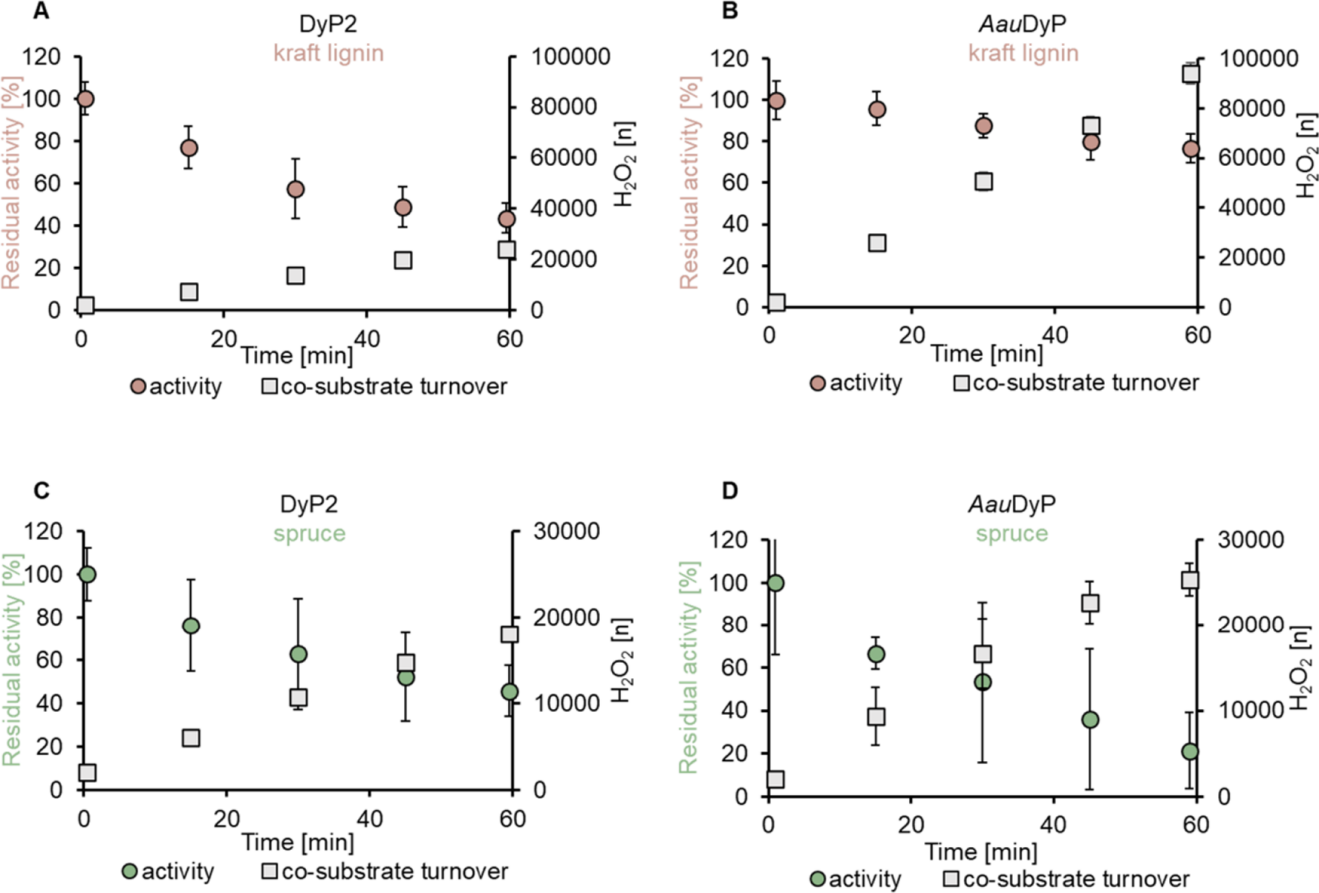
Stability of bacterial and fungal dye-decolorizing peroxidase on kraft lignin and untreated spruce. For each measurement, the sensor was first calibrated by stepwise titration of 40 μM H_2_O_2_ to a final concentration of 200 μM while rotating at 2000 rpm in the 100 g L^–1^ substrate mixture at pH 4.5. The reaction was initiated by the addition of 0.1 μM of the enzyme. After complete conversion of H_2_O_2_, 200 μM of fresh H_2_O_2_ was repeatedly added to the reaction mixture for 1 h. The average of triplicates is reported every 15 min. The residual activity of (A) DyP2 and (B) *AauDyP* on kraft lignin was 40 and 80% of its initial activity, respectively. In 1 h, DyP2 performed 24,000 turnovers of H_2_O_2_, whereas *AauDyP* performed 94,000 turnovers of H_2_O_2_. The residual activity of (C) DyP2 and (D) *AauDyP* on spruce was similar, in line with the similar performance in H_2_O_2_ turnovers.

To evaluate the turnover stability, defined as the comparison of enzymes for their ability to continue and maintain catalysis during an extended time period, the activity of bacterial and fungal dye-decolorizing peroxidases was measured on kraft lignin and untreated spruce. Upon reaching 0 μM in the H_2_O_2_ concentration, indicative of the full conversion of cosubstrate by the peroxidase, 200 μM H_2_O_2_ was titrated repeatedly to the reaction mixture for 1 h (Figures S7 and S8). The residual activities were determined from the linear regression of freshly added H_2_O_2_ time trace measurements. After 1 h, DyP2 retained 40% of its initial activity (Figure 4A), whereas *Aau*DyP maintained 80% of its initial activity (Figure 4B) when utilizing kraft lignin as a substrate. The larger substrate availability for *Aau*DyP, oxidizing both phenolic and nonphenolic compounds, may have prevented self-inactivation by the cosubstrate, allowing for a continuous peroxidase reaction cycle. Huang and colleagues improved the H_2_O_2_ stability of *Irpex lacteus* F17 dye-decolorizing peroxidase.^40^ It would indeed be intriguing to investigate the activity of the engineered enzyme with a H_2_O_2_ sensor on complex lignocellulosic substrates. Such studies can provide valuable insights for applying dye-decolorizing peroxidases in lignocellulose degradation processes.

Bacterial DyP2 performed 24000 turnovers of H_2_O_2_ (Figure 4A) and retained 40% of its initial activity, whereas the high catalytic rates of *Aau*DyP (Figure 3B) combined with the retention of 80% residual activity enabled the enzyme to achieve 94000 turnovers of H_2_O_2_ (Figure 4B) when kraft lignin was used as substrate. In the common peroxidase reaction (Figure 1), two substrate molecules donating one electron each or one substrate molecule donating two electrons are oxidized with the reduction of one H_2_O_2_ to H_2_O.^16,41^ Hence, theoretically, the reaction of fungal or bacterial DyP with kraft lignin results in 188,000 and 48,000 oxidized product molecules, respectively, showcasing the remarkable stability of both enzymes under turnover conditions. For H_2_O_2_-driven cytochrome P450 and chloroperoxidase, lower turnover numbers were reported (<1000^42,43^ and approximately 7000,^44^ respectively). It stands to reason that these numbers may be underestimated due to the assay conditions and could perhaps be similarly increased. Another study showed that addition of poly(ethylene glycol) to the enzymatic reaction increased total turnovers of various peroxidases.^45^ Thus, the findings reported here hold particular significance for industrial applications.

In earlier studies, varying concentrations of H_2_O_2_ have been employed for DyP activity determination, but H_2_O_2_ was generally added in one batch. For instance, the DyP reaction on lignocellulose or extracted lignin material was performed with 100 μM H_2_O_2_,^24,46^ which likely did not exploit the catalytic potential of the enzyme. Similarly, the reaction of *Pseudomonas fluorescens* DyP with wheat straw using 1000 μM H_2_O_2_^23^ resulted in one peak in HPLC, suggesting a low number of oxidized degradation products. Pupart and colleagues supplemented the overnight incubation of *Streptomyces coelicolor* A3(2) DyP on organosolv lignin with 2000 μM H_2_O_2_, possibly causing enzyme inactivation due to the high amount of H_2_O_2_.^22^ Inactivation of *Aau*DyP and other DyP by H_2_O_2_ was reported previously.^23,46−49^*In vivo*, H_2_O_2_ can be continuously supplied by FAD-dependent oxidases or laccases,^50^ and their interplay with peroxidases has been demonstrated *in vitro.*(51−53) Co-immobilization with glucose oxidase even improved operational stability of peroxidases,^44^ which again may explain the high turnover of H_2_O_2_ upon continuous supply of lower concentrations of H_2_O_2_.^54^

As mentioned above, the production of H_2_O_2_*in vivo* is controlled by a number of different oxidases. This means that the amount of 200 μM H_2_O_2_, which is a standard reaction condition in this study, is not representative of the natural conditions. Therefore, the DyP2 reaction on 100 g L^–1^ kraft lignin was performed with 50 μM H_2_O_2_ by extending the reaction time to 3 h. Figure S9 shows that when 50 μM H_2_O_2_ was titrated over 3 h, DyP2 performed 22500 turnovers of H_2_O_2_ while remaining 77% active. In comparison, when 200 μM H_2_O_2_ was added over 1 h, the enzyme performed 24000 turnovers of H_2_O_2_ and remained 40% active (Figure 4A). Thus, the H_2_O_2_ concentration plays an important role in achieving high stability under turnover conditions.

After 24000 turnovers of H_2_O_2_ by the bacterial peroxidase, the insoluble fraction of kraft lignin (Figure 4A) was separated and analyzed by gel permeation chromatography. No significant difference between the enzymatically treated samples and the control samples was observed (Figure S10). This could be explained by the preferential consumption of partially solubilized low-molecular-weight aromatic compounds rather than reaction on the high-molecular-weight fraction, or by a rapid repolymerization of the formed radicals.^28,55,56^

On spruce, the measurements with bacterial and fungal DyP revealed similar behavior (Figure 4C,D), consistent with the earlier measured catalytic rates (Figure 3). Both enzymes remained around 30–40% active and achieved comparable H_2_O_2_ turnovers (DyP2 18,000, *Aau*DyP 25,000). To understand if the decrease in catalytic rate of the enzyme resulted from self-inactivation or H_2_O_2_ inactivation due to substrate depletion, a fresh amount of *Aau*DyP was added to the reaction mixture toward the end of the reaction (Figure S11). The additional enzyme dose did not lead to a significant increase in catalytic rates (from 5.8 to 6.3 s^–1^), suggesting substrate depletion.

To investigate this further, the bacterial DyP2 reaction was carried out on 1 g L^–1^ kraft lignin to achieve rapid substrate depletion but still allow the conversions to occur under saturated conditions. A significant decrease (from TN 38 ± 1 s^–1^ to TN 5 ± 1 s^–1^) in the catalytic rates of DyP2 was observed after the third titration of 200 μM H_2_O_2_ (Figure S12A). When 100 μL of 100 g L^–1^ kraft lignin was freshly added to the reaction mixture, the catalytic rate did not improve, indicating that the enzyme was inactive. Subsequently, when a fresh amount of DyP2 (0.1 μM) was added, the catalytic rate returned to its initial value, indicating that the fresh substrate was sufficient to reach saturated conditions. Furthermore, in Figure S12B, when the low catalytic rates of DyP2 were reached after the third titration of 200 μM H_2_O_2_, fresh enzyme was added to the reaction mixture before the new substrate. Again, no improvement in the catalytic rate by the new enzyme was observed, indicating that the preferred substrate had already been consumed by the initially added enzyme. After the addition of fresh kraft lignin, the newly added enzyme performed at the maximum turnover number.

It is known from the literature that H_2_O_2_ causes inactivation of peroxidases.^33,34^ To verify that the freshly added DyP2 in Figure S12B was not inactivated by residual H_2_O_2_ and to understand the rough time course when inactivation occurs, DyP2 was incubated with 200 μM H_2_O_2_ in the reaction buffer without any substrate for 10 min (Figure S13). After the addition of 100 μL of 100 g L^–1^ kraft lignin, the measured catalytic rate was 17 s^–1^, which is about half of the previously determined rate (TN 38 ± 1 s^–1^), suggesting that even 200 μM H_2_O_2_ has an effect on the enzyme activity. Thus, reducing compounds must be present to avoid inactivation by the oxidant.

The same experiments as shown in Figure S12 could not be carried out with spruce as a substrate, because the consistency of the 100 g L^–1^ suspension made it challenging to supplement a small volume with a high substrate load. Nevertheless, Figure S14 shows that the higher the concentration of spruce in the reaction, the more H_2_O_2_ is converted by the enzyme and the slower the decrease in the catalytic rates is. Thus, dye-decolorizing peroxidases are inactivated as a consequence of unconsumed H_2_O_2_, which is caused by substrate depletion.

### 2.3 Product Analysis of the Bacterial Dye-Decolorizing Peroxidase on Kraft Lignin

**Figure 5:**
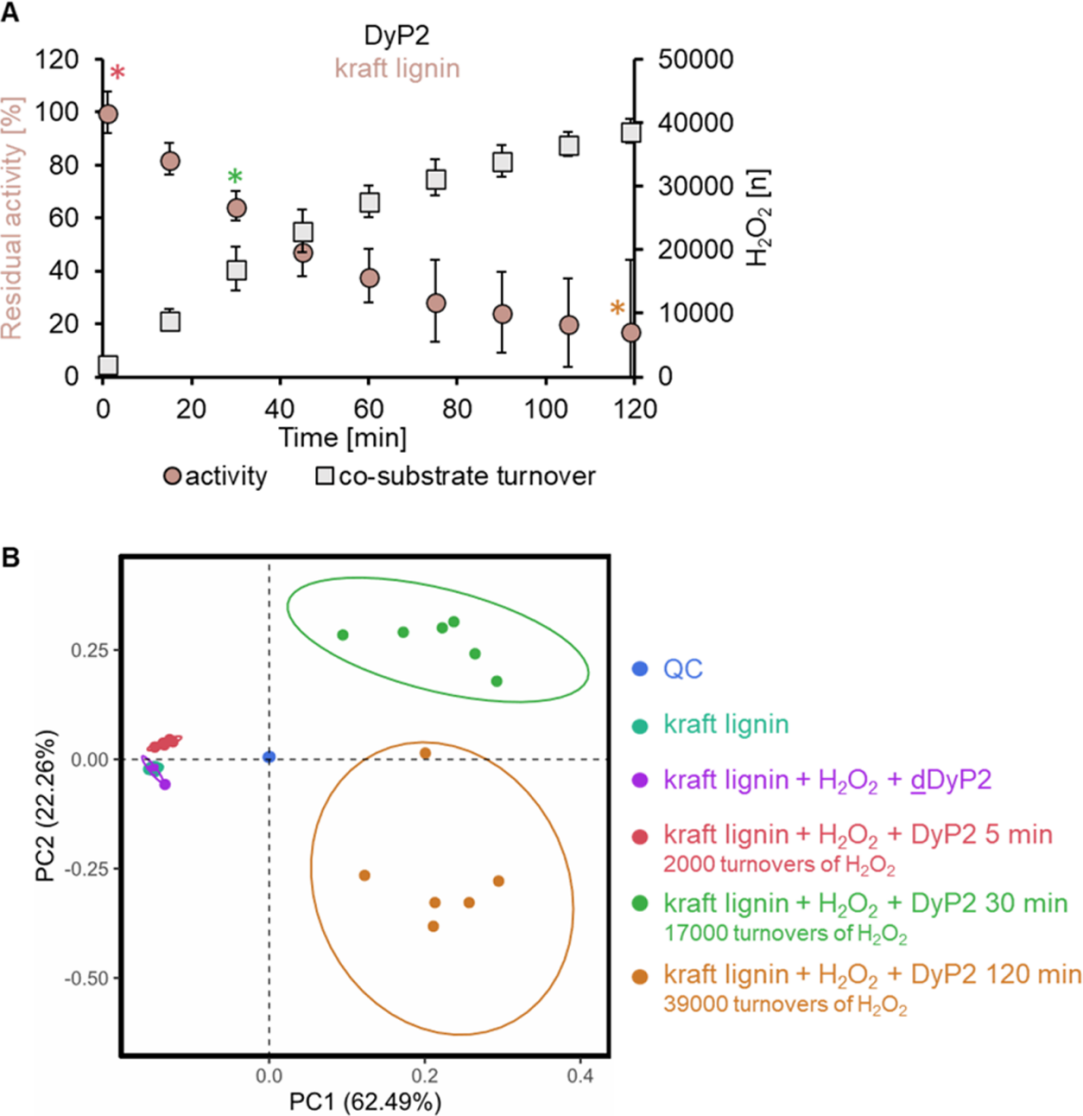
Verification of DyP2 activity on kraft lignin. (A) Stability of DyP2 on kraft lignin after 2 h. The residual activity and H_2_O_2_ turnovers were reported every 15 min in six replicates. DyP2 had lost 80% of its activity after 2 h but achieved 39,000 turnovers of H_2_O_2_. The samples for nontargeted mass spectrometry were collected at 5, 30, and 120 min as marked with asterisks (*). (B) Nontargeted high-resolution mass spectrometry of DyP2 reaction products. In the principal component analysis, the control samples (kraft lignin, kraft lignin with H_2_O_2_, and denatured DyP2, dDyP2) were grouped differently compared to the 5, 30, and 120 min sample points representing the variation in composition. All of the samples were pooled for system quality control (QC). Ellipses around sample clusters represent 95% confidence, colored dots are acquisition data (*n* = 6).

Considering that DyP2 retained approximately 40% of its activity on kraft lignin after 1 h (Figure 4A), the experiment was extended to 2 h (Figure 5) to maximize product formation for further analysis. At the 2 h time point, the remaining activity of DyP2 was 20% of its initial activity after a total of 39000 turnovers of H_2_O_2_ (Figure 5A). Samples, collected at 5, 30, and 120 min time points, were subjected to nontargeted mass spectrometry analysis. The 120 min samples were observed to be darker in color compared to the control (Figure S15).

Principal component analysis (PCA) has been previously used to investigate enzymatically treated lignin;^24^ thus, this analysis was conducted to show the differences between controls and enzymatically treated samples (Figure 5B). After blank filtration, 36 features were left and utilized in the PCA, which covered 84.8% of the total variance within the first two principal components (PC1 and PC2). The samples of kraft lignin only and kraft lignin with H_2_O_2_ and denatured DyP2 were grouped together, indicating minimal differences in the product profiles. The 5 min time points (2000 turnovers of H_2_O_2_) grouped well together and slightly separated from the controls, indicating changes in the product profile caused by the oxidative activity of bacterial DyP2. Notably, at later time points, the samples after 30 min (17,000 turnovers of H_2_O_2_) and 120 min (39,000 turnovers of H_2_O_2_) showed replicates grouped together and clearly separated from the other conditions, indicating distinct changes in compounds that were not present in the control samples. The exact compounds in which DyP2 was active could not be identified by this approach and was not the aim of this work. The pooled quality control (QC) samples all overlapped and sat between controls and time points, validating the analysis. Nontargeted high-resolution mass spectrometry revealed a relationship between consumption of H_2_O_2_ and product formation, verifying the ability of the H_2_O_2_ sensor to measure peroxidase activity on heterogeneous lignocellulosic substrates.

## 3 Conclusions

Bacterial and fungal dye-decolorizing peroxidases were assayed on lignocellulosic materials using a H_2_O_2_ sensor. The findings revealed catalytic differences in bacterial and fungal DyP on various substrates accompanied by remarkable turnover numbers of H_2_O_2_, particularly when lower concentrations of H_2_O_2_ are added in a continuous fashion. The stability under turnover conditions depends on the oxidant (H_2_O_2_) and available reducing compounds (substrate); the enzymes become inactivated by the cosubstrate upon substrate depletion. The amount of consumed H_2_O_2_ correlates with product formation as shown by mass spectrometry analysis, underscoring the usefulness of the H_2_O_2_ sensor in peroxidase activity determination.

## 4 Experimental Section

### 4.1 Materials

Kraft lignin, lignosulfonate, Nafion, potassium ferricyanide, ferric chloride, hydrogen peroxide, potassium chloride, sodium acetate, acetic acid, 2-amino-2-(hydroxymethyl)-1,3-propandiol (Tris base), imidazole, magnesium sulfate, calcium chloride, glucose, tryptone, yeast extract, Lab Lemco powder, and chitin were purchased from Sigma-Aldrich (Darmstadt, Germany). Thiostrepton was purchased from Merck Millipore (Darmstadt, Germany). Xylan, mannan, and curdlan were purchased from Megazymes (Bray, Ireland). Restriction enzymes were obtained from New England Biolabs (Ipswich, MA).

### 4.2 Protein Production and Purification

Bacterial dye-decolorizing peroxidase gene from *Amycolatopsis* 75iv2 (WP_020421762.1) was expressed as previously published.^57^ Briefly, the *E. coli* cloning plasmid was constructed using Gibson Assembly (New England Biolabs). *E. coli* plasmid pUC-P_vsi_^58^ was linearized with *Pst*I, incubated with the inset containing the overhangs, and transformed into chemically competent *E. coli* JM109. pUC-P_vsi_-DyP2 and the *S. lividans* plasmid pIJ486 were both digested with *Hind*III and *Xba*I, ligated with T4 ligase, and transformed into *S. lividans* TK24 protoplasts as described in ref (59). *S. lividans* spores were used to produce the enzyme in 1 L baffled flasks, were harvested by centrifugation, and the cell pellet was resuspended in buffer A (50 mM Tris 300 mM NaCl pH 7.4), sonicated, centrifuged, and the filtrate was purified using affinity chromatography.

Bacterial dye-decolorizing peroxidase from *E. coli* was a generous gift from Vera Pfanzagl, Department of Chemistry, BOKU University. The gene encoding a dye-decolorizing peroxidase from *Auricularia auricula-judae* was expressed as previously reported.^47^

### 4.3 Lignocellulosic and Carbohydrate Material Preparation

Birch (Rahula, Estonia), spruce (Vienna, Austria), poplar (Vienna, Austria), mate tea (Paraguay) left-over residues, wheat straw (Vienna, Austria), sugarcane (Vienna, Austria), and corn (Vienna, Austria) were ground using benchtop miller and separated with molecular sieves (Haver and Boecker, Oelde, Germany). The achieved particle size was smaller than 125 μm. For enzyme assays, 100 g L^–1^ of each lignocellulosic material was resuspended in 50 mM sodium acetate pH 4.5 buffer and incubated at 30 °C 110 rpm overnight. Additional substrate concentrations of 1, 2, 5, 10, and 25 g L^–1^ were employed for the determination of catalytic rates on kraft lignin and spruce. For the carbohydrate test, hemicelluloses xylan (100 g L^–1^), mannan (50 g L^–1^), curdlan (50 g L^–1^), and chitin (50 g L^–1^) were also resuspended in 50 mM sodium acetate pH 4.5 buffer and incubated at 30 °C 110 rpm overnight. Mannan, curdlan, and chitin were used in lower substrate concentrations due to the high viscosity.

### 4.4 Preparation of the Rotating Disk Electrode (RDE) for the H_2_O_2_ Sensor

The H_2_O_2_ sensor is based on a gold rotating disk electrode modified with a thin film of deposited Prussian blue by using cyclic voltammetry. The electrodes were prepared as previously described.^32^ Briefly, the electrode was submerged into 10 M NaOH for 1 min and then rinsed with deionized H_2_O. Next, the gold RDE was polished with aqueous alumina particles (0.05 μm) on MicroCloth (Buehler, Lake Bluff, IL), and residual polishing particles were removed by sonication in a water bath for 5 min. Prussian blue was deposited on the electrode surface by performing cyclic voltammetry in a solution containing 1 mM FeCl_3_, 1 mM K_3_[Fe(CN)_6_], 0.1 M KCl, and 0.1 M HCl by 12 potential sweep cycles between 600 and 900 mV vs SHE at a scan rate of 20 mV s^–1^. Activation of the deposited Prussian blue layer was done by cycling the electrode 20 times between 160 and 590 mV vs SHE at a scan rate of 50 mV s^–1^ in a solution containing 100 mM KCl and 100 mM HCl. After activation, the H_2_O_2_ sensor was air-dried and coated with 5 μL of Nafion (Sigma-Aldrich). The H_2_O_2_ sensor was stored overnight under an ambient atmosphere.

### 4.5 The Enzymatic Reaction in RDE Setup

The Prussian-blue-deposited electrode was immersed in the reaction mixture and rotated at a speed of 2000 rpm. The mixture, in a total of 4 mL, contained 225 mM KCl and 100 or 50 g L^–1^ substrates. Measurements to determine the catalytic rate on kraft lignin and spruce were also done at 1, 2, 5, 10, and 25 g L^–1^. The electrode was calibrated stepwise with 40 μM H_2_O_2_ to a final concentration of 200 μM while measuring at the potential of 200 mV vs the standard hydrogen electrode (SHE). The data were collected every 0.08 s at room temperature. The stabilization of the current at each titration step was recorded for 15–25 s and served as an indicator of the absence of side reactions. Subsequently, the reaction was initiated with 0.1 μM enzyme. To determine the initial rates, linear regression was performed on the data collected between 40 and 50 s from the beginning of the H_2_O_2_ conversion. H_2_O_2_ turnovers were calculated as *n* = *c*(H_2_O_2_)/*c*(DyP2), where *c* is the concentration. For stability measurements, after complete conversion of the H_2_O_2_, fresh amounts of cosubstrate were added repeatedly for 1, 2, or 3 h. Residual activities were calculated from the linear regression of the new catalytic cycle upon fresh addition of H_2_O_2_ using data points between 40 and 50 s. Residual activities and H_2_O_2_ turnovers are reported every 15 min.

### 4.6 High-Resolution Mass Spectrometry

The samples of 5, 30, and 120 min from the 2 h kraft lignin time course experiment were filtered through 10000 Da Amicon (Merck Millipore) prior to injection. A volume of 5 μL of the sample solution was directly injected into an LC-ESI-HRMS system. A UHPLC (Agilent 1290 Infinity II UPLC; Santa Clara, CA) was used for the separation of the analytes with a gradient 0–12 min 1 to 18% B, 12–20 min 18–60% B, 20–21 min 60–90% B, 21–22 min 90% B, 22–22.1 min 1% B, 22.1–30 min 1% B, where A is H_2_O + 0.1% formic acid (FA) and B is acetonitrile with 0.1% FA. The stationary phase was an Acquity UPLC HSS T3 (C18) column (100 Å, 1.8 μm particle size, 2.1 mm I.D. x 150 mm length; Waters, Milford, MA) and a flow rate of 100 μL min^–1^ was applied. Detection was performed with a Q-TOF instrument (Agilent Series 6560 LC-IMS-QTOFMS) equipped with the Jetstream ESI source in negative-ion mode (range: 50–1700 *m*/*z*). Instrument calibration was performed using an ESI calibration mixture (Agilent). A QC sample was composed of 30 samples pooled eudiometrically.

LC-HRMS data was preprocessed in MS DIAL (version 4.9.221218). Kraft lignin with H_2_O_2_ and denatured DyP2 (kraft lignin+H_2_O_2_+dDyP2) control samples were used for blank filtrating (<20% blank peak area threshold). Locally weighted scatterplot smoothing regression was used for QC normalization of samples. Further preprocessing parameters can be found in Table S1. Preprocessed data was exported in R (version 4.3.2), centered, and auto-scaled, and a PCA was performed.

### 4.7 Gel Permeation Chromatography–Multiangle Light Scattering (GPC-MALS)

Samples were dissolved in DMSO/LiBr (0.5%) to achieve a concentration of 10 g mL^–1^. Prior to GPC analysis, the solutions were filtered through a 0.45 μm PTFE syringe filter. The analysis of molecular weight distribution was done by means of MALS 785 nm detector in accordance with ref (60). The specific refractive index increment (d*n*/d*c*)_μ_ of 0.150 was used in molar mass calculations.

## Supporting Information Available

Time trace measurements of DyP2 on lignocellulosic substrates (Figure S1); catalytic rates of DyP2 on different kraft lignin concentrations (Figure S2); catalytic rates of DyP2 on different concentrations of spruce (Figure S3); enzyme-independent reaction between lignosulfonate and H_2_O_2_ (Figure S4); time trace measurements of DyP2 on carbohydrate substrates (Figure S5); time trace measurements of *E. coli* DyPA on kraft lignin (Figure S6); continuous conversion of H_2_O_2_ by fungal and bacterial DyP on kraft lignin (Figure S7); continuous conversion of H_2_O_2_ by fungal and bacterial DyP with untreated spruce as substrate (Figure S8); continuous conversion of a lower concentration of H_2_O_2_ (50 μM) by fungal and bacterial DyP on kraft lignin (Figure S9); GPC-MALS of insoluble kraft lignin after enzymatic reaction for 1 h (Figure S10); inactivation of fungal DyP by substrate depletion (Figure S11); inactivation of bacterial DyP2 on kraft lignin (Figure S12); effect of H_2_O_2_ on bacterial DyP2 (Figure S13); inactivation of bacterial DyP2 on untreated spruce (Figure S14); kraft lignin samples after 2 h of enzyme reaction (Figure S15); and preprocessing parameters in MS DIAL (Table S1). (PDF)

## Abbreviations

DyP: dye-decolorizing peroxidase
UV/vis: ultraviolet–visible light
MS: mass-spectrometry
NMR: nuclear magnet resonance
FT-IR: Fourier transform infrared spectroscopy
GPC: gel penetration chromatography
ABTS: 2,2′-azino-bis(3-ethylbenzothiazoline-6-sulfonic acid)
DMP: 2,6-dimethoxyphenol
GGE: guaiagylglycerol β-guaiacyl ether
VGE: veratrylglycerol-β-guaiacyl ether
LPMO: lytic polysaccharide monooxygenase
TN: turnover number
DyP2: DyP from *Amycolatopsis* 75iv2
*Aau*DyP: DyP from *Auricularia auricula-judae*
FAD: flavin adenine dinucleotide
PC(A): principal component (analysis)
QC: quality control
