# Supplementary material for: Dye-Decolorizing Peroxidases Maintain High Stability and Turnover on Kraft Lignin and Lignocellulose Substrates
Source: ACS Omega. 2024 Oct 31;9(45):45025–34. doi: 10.1021/acsomega.4c05043 (PMC11561623; doi:10.1021/acsomega.4c05043)
Supplement: Supplementary file 1 — ao4c05043_si_001.pdf [file ao4c05043_si_001.pdf]

Supporting Information for

Dye-decolourising peroxidases maintain high stability and turnover on Kraft lignin and lignocellulose substrates

Silja Välimets[1,2], Lorenz Schwaiger[1,2], Alexandra Bennett[3], Daniel Maresch[4], Roland Ludwig[1,2], Stephan Hann[2,3], Dolores Linde[5], Francisco Javier Ruiz-Dueñas[5], Clemens Peterbauer[1,2]

[1] Department of Food Science and Technology, Institute of Food Biotechnology, BOKU University, Muthgasse 11, 1190, Vienna, Austria

[2] Doctoral Programme BioToP- Biomolecular Technology of Proteins, BOKU University, Muthgasse 18, 1190, Vienna, Austria

[3] Department of Chemistry, Institute of Analytical Chemistry, BOKU University, Muthgasse 18, 1190, Vienna, Austria

[4] Core Facility Mass-spectrometry, BOKU University, Muthgasse 11, 1190, Vienna, Austria

[5] Centro de Investigaciones Biológicas Margarita Salas (CIB), Consejo Superior de Investigaciones Científicas (CSIC), Ramiro de Maeztu 9, 28040, Madrid, Spain

This file contains:

Supplementary Figures S1 to S15 page 2 to page 12

Supplementary Table SM1 page 13 to 14

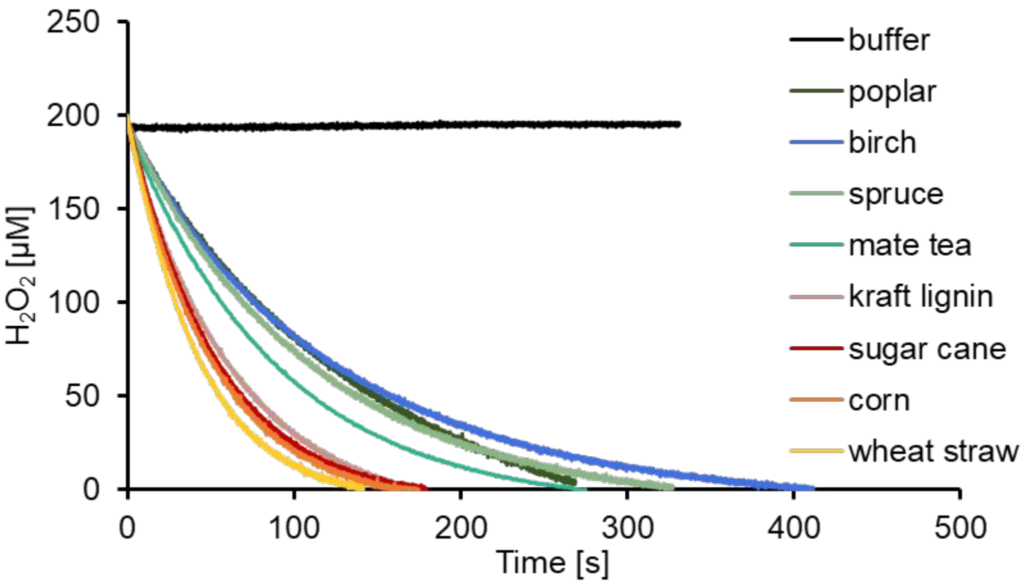

Figure S1: Time trace measurements of DyP2 from *Amycolatopsis* 75iv2 on various lignocellulosic substrates. For each measurement the sensor was first calibrated by stepwise titration of 40 µM H_2O_2 to a final concentration of 200 µM while rotating at 2000 rpm in the 100 g L^{-1} substrate mixture at pH 4.5. The reaction was initiated by the addition of 0.1 µM of DyP2. The measured currents were converted to H_2O_2 concentrations (µM) over time (s). Reactions were conducted in triplicates, but only one replicate is shown. Reaction buffer was used as a negative control.

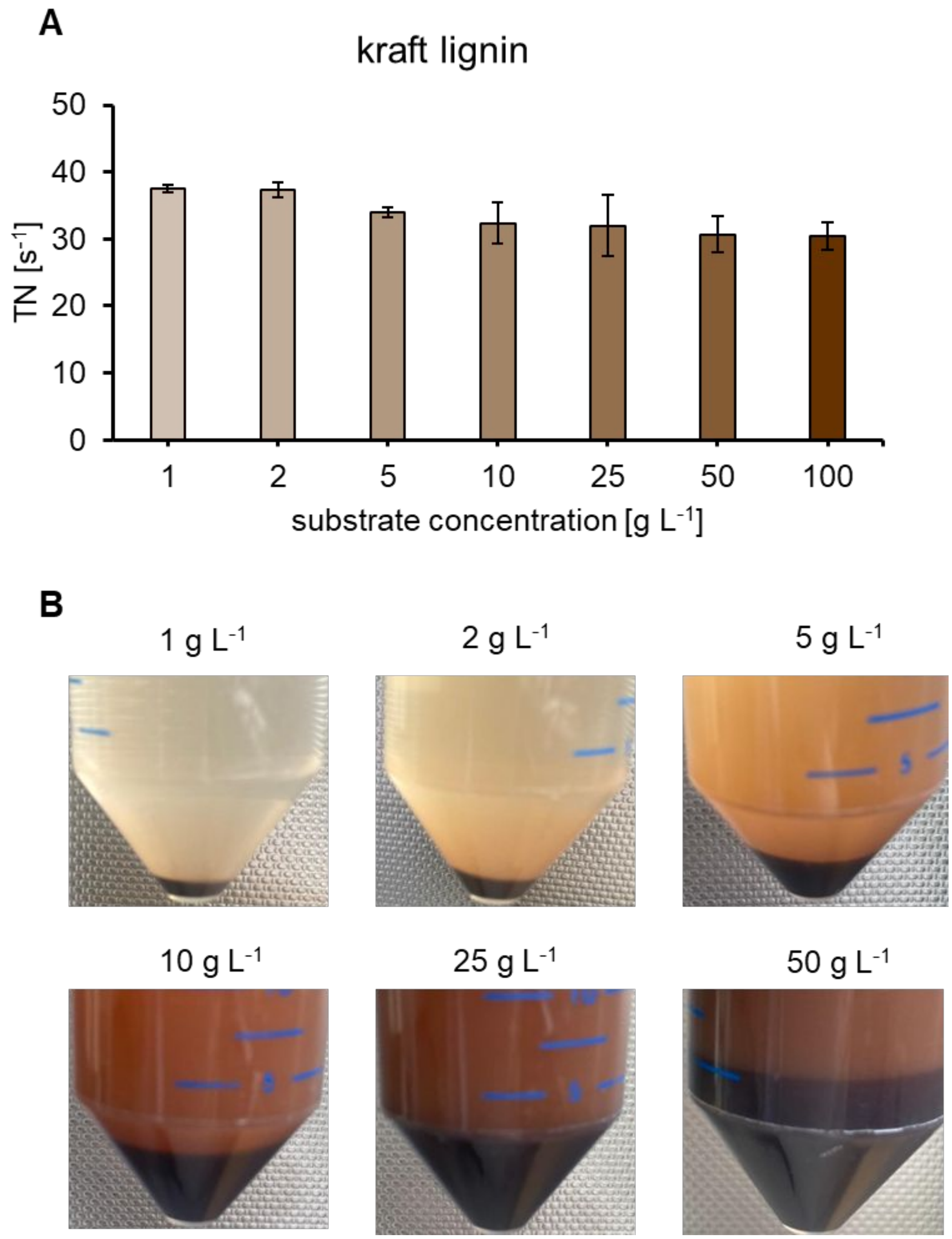

Figure S2: The catalytic rates of bacterial DyP2 on different kraft lignin concentrations. A) For each measurement the sensor was first calibrated by the stepwise addition of 40 µM H_2O_2 to a final concentration of 200 µM while rotating at 2000 rpm in substrate suspension at pH 4.5. The reaction was initiated by the addition of 0.1 µM of DyP2. The measured currents were converted to H_2O_2 concentrations (µM) over time (s). The determined catalytic rates indicated that the reactions were running at saturated conditions. **B)** Kraft lignin suspensions at different concentrations. The suspensions were vigorously vortexed to ensure uniform distribution of the sediment.

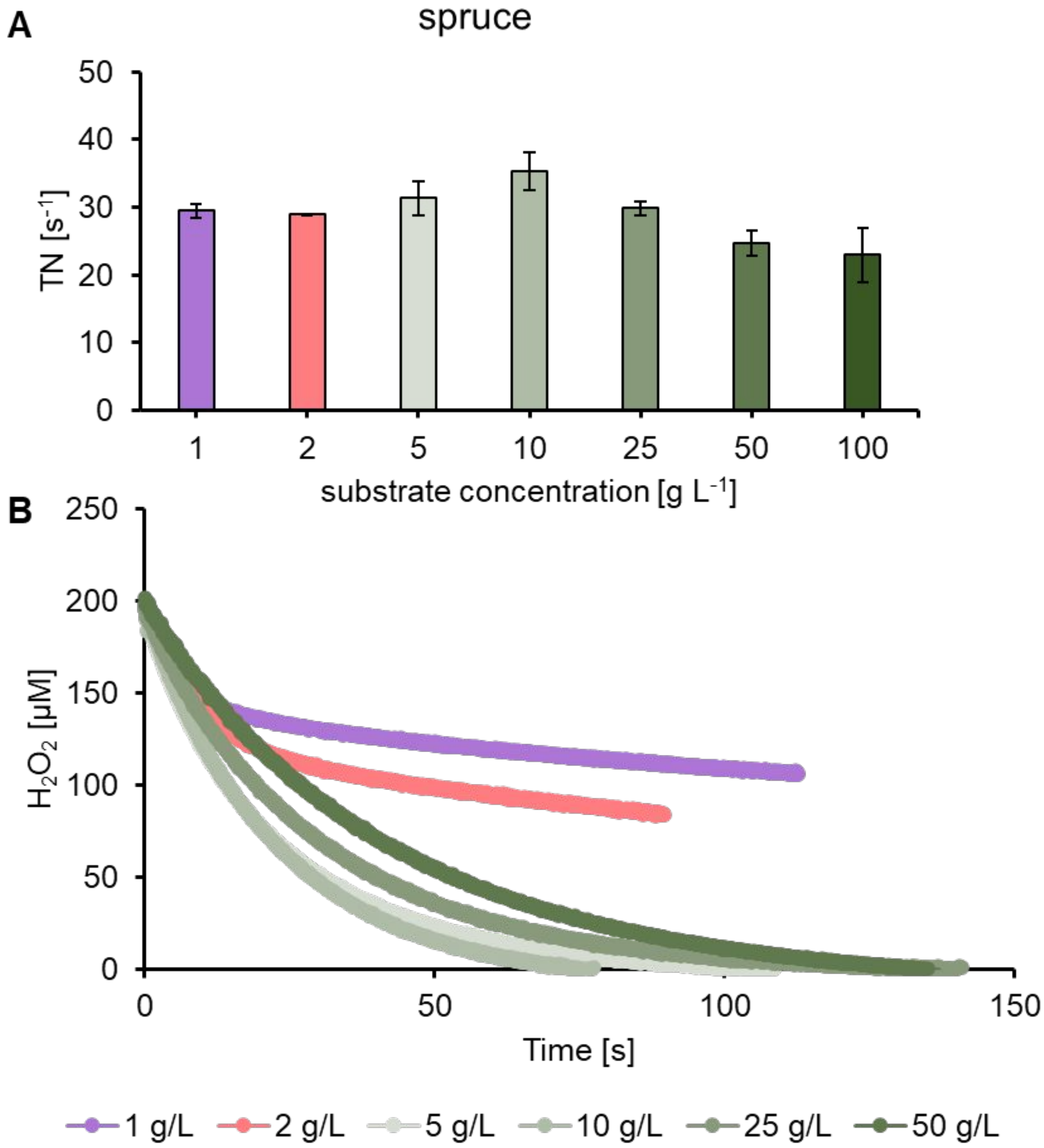

Figure S3: The catalytic rates of bacterial DyP2 on different concentrations of spruce. **A)** For each measurement the sensor was first calibrated by the stepwise titration of 40 µM H_2O_2 to a final concentration of 200 µM while rotating at 2000 rpm in the substrate suspension at pH 4.5. The reaction was initiated by the addition of 0.1 µM of DyP2. The measured currents were converted to H_2O_2 concentrations (µM) over time (s). The determined catalytic rates indicated that each reaction was running at saturated conditions. **B)** Time trace measurements of DyP2 acting on different concentrations of spruce suspension. Spruce 1 g L^{-1} and 2 g L^{-1} suspensions did not reach the baseline again, indicating that these suspensions did not contain enough substrate to convert all the 200 µM H_2O_2, whereas 5, 10, 25, 50 g L^{-1} reached the baseline.

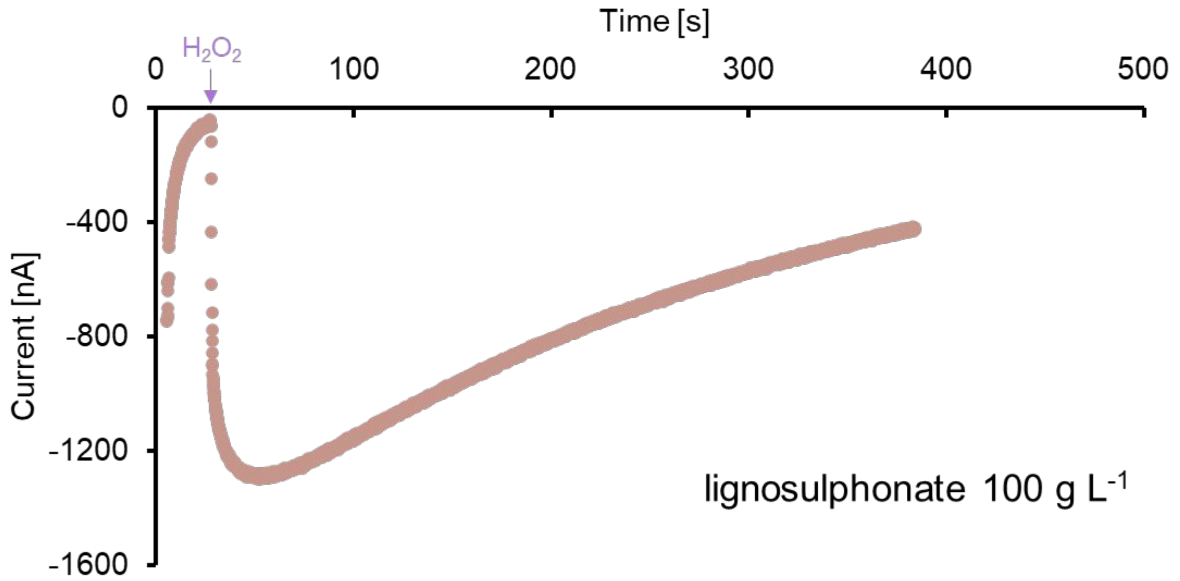

Figure S4: Enzyme independent reaction between the substrate and H_2O_2. 200 µM of H_2O_2 was added directly to the lignosulphonate suspension (100 g L^{-1}) without stepwise calibration and a rapid decrease of H_2O_2 was detected. After 100 s the current continued to decrease and was not stabilised indicating H_2O_2 reactivity with unknown compounds in the lignosulphonate suspension.

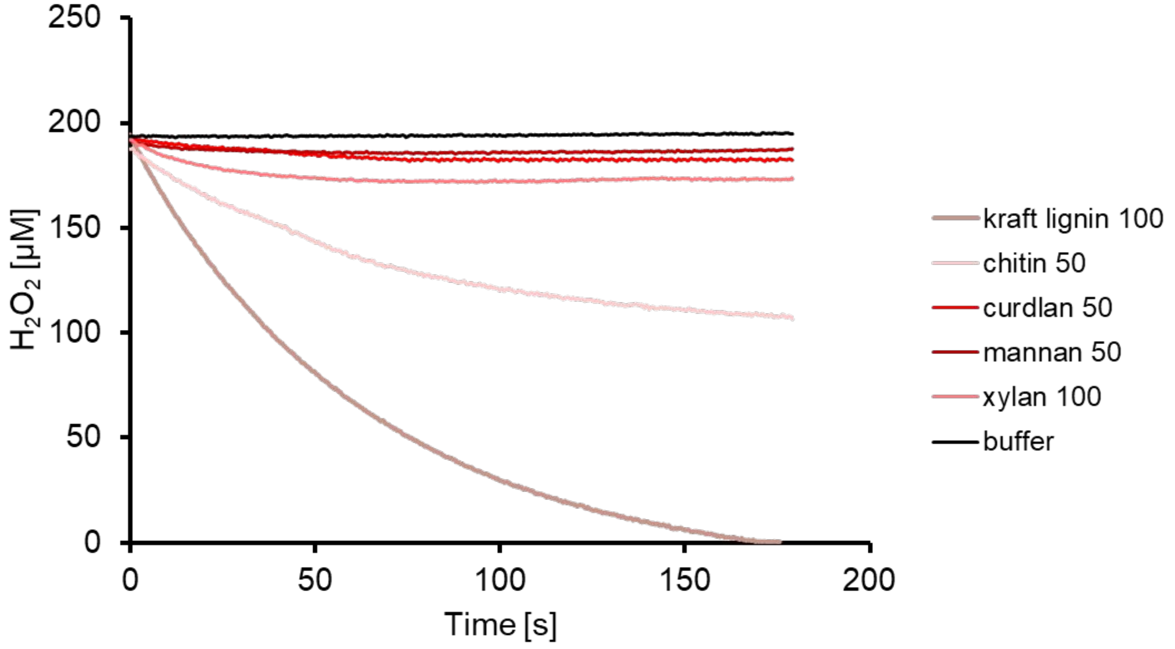

Figure S5: DyP2 time trace measurements using carbohydrates as substrates. For each measurement the sensor was first calibrated by stepwise titration of 40 µM H_2O_2 to a final concentration of 200 µM while rotating at 2000 rpm in the defined substrate suspension at pH 4.5. 50 g L^{-1} of chitin, curdlan, mannan and xylan were prepared due to the high viscosity of the samples. The reaction was initiated with the addition of 0.1 µM of DyP2. The measured currents were converted to H_2O_2 concentrations (µM) over time (s). Buffer alone was used as a negative control and kraft lignin was used as a positive control. DyP2 was not active with curdlan, mannan and xylan. Background reactivity was measured in the presence of chitin, but notably H_2O_2 consumption did not

reach to 0 µM as it did for kraft lignin suggesting a minor amount of substrate reacting with the enzyme, most likely a contamination with unknown phenolic compounds. The chitin used here was of technical grade purity.

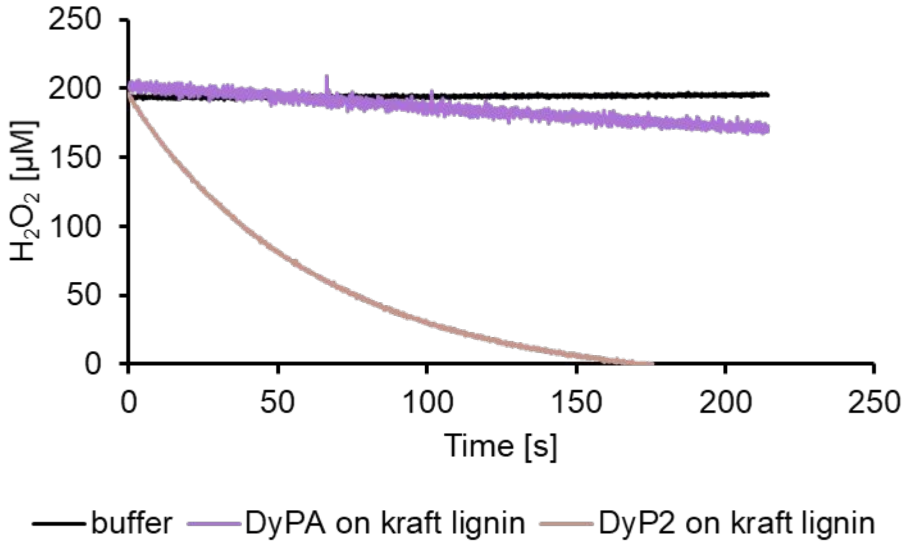

Figure S6: **The time trace measurements of DyPA from *E. coli* using kraft lignin as a substrate.** For each measurement the sensor was first calibrated by stepwise titration of 40 µM H_2O_2 to a final concentration of 200 µM while rotating at 2000 rpm in the kraft lignin suspension at pH 4.5. The reaction was initiated with the addition of 0.1 µM of DyP2. The measured currents were converted to H_2O_2 concentrations (µM) over time (s). Buffer alone was used as a negative control and DyP2 reaction with kraft lignin was used as a positive control. DyPA showed no H_2O_2 conversion in 200 s compared to DyP2, which completely converted H_2O_2.

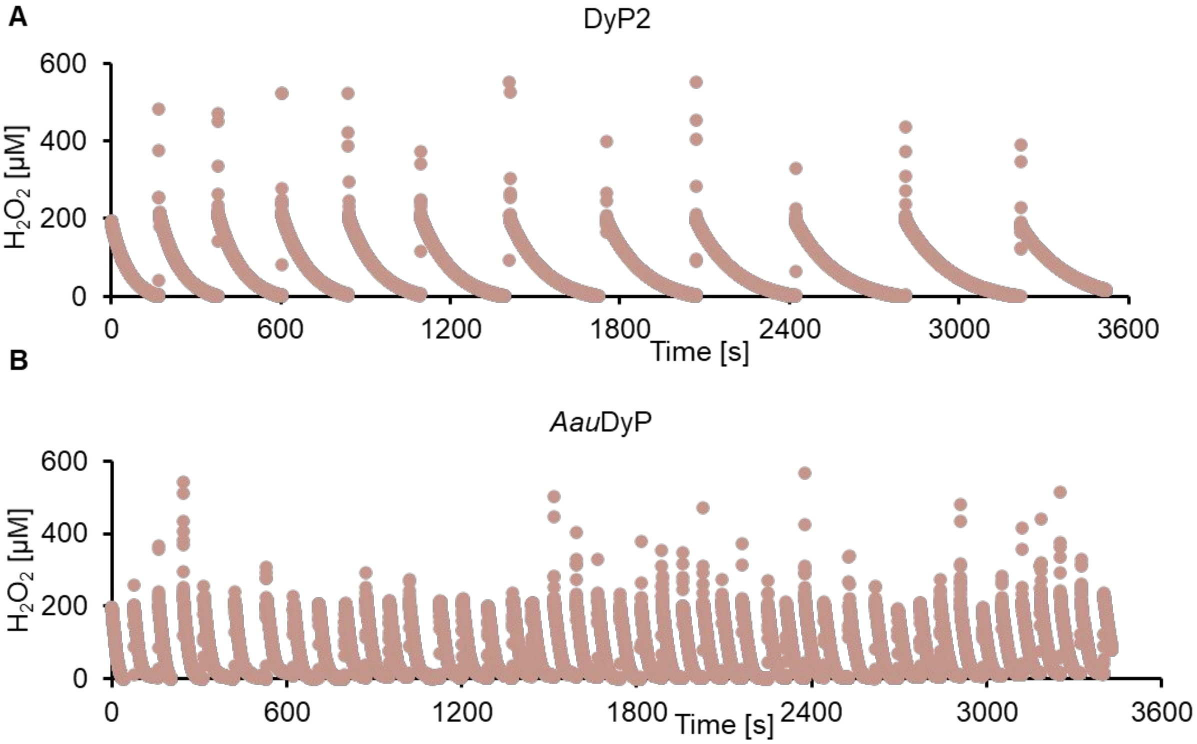

Figure S7: The continuous conversion of H_2O_2 by bacterial and fungal dye-decolourizing peroxidases on kraft lignin. For each measurement the sensor was first calibrated by the stepwise titration of 40 µM H_2O_2 to a final concentration of 200 µM while rotating at 2000 rpm in the 100 g L^{-1} substrate mixture at pH 4.5. The reaction was initiated by the addition of 0.1 µM of the enzyme. After complete conversion of H_2O_2, 200 µM of fresh H_2O_2 was repeatedly added to the reaction mixture for one hour. The H_2O_2 conversion of **A)** bacterial DyP2 and **B)** fungal *Aau*DyP on kraft lignin.

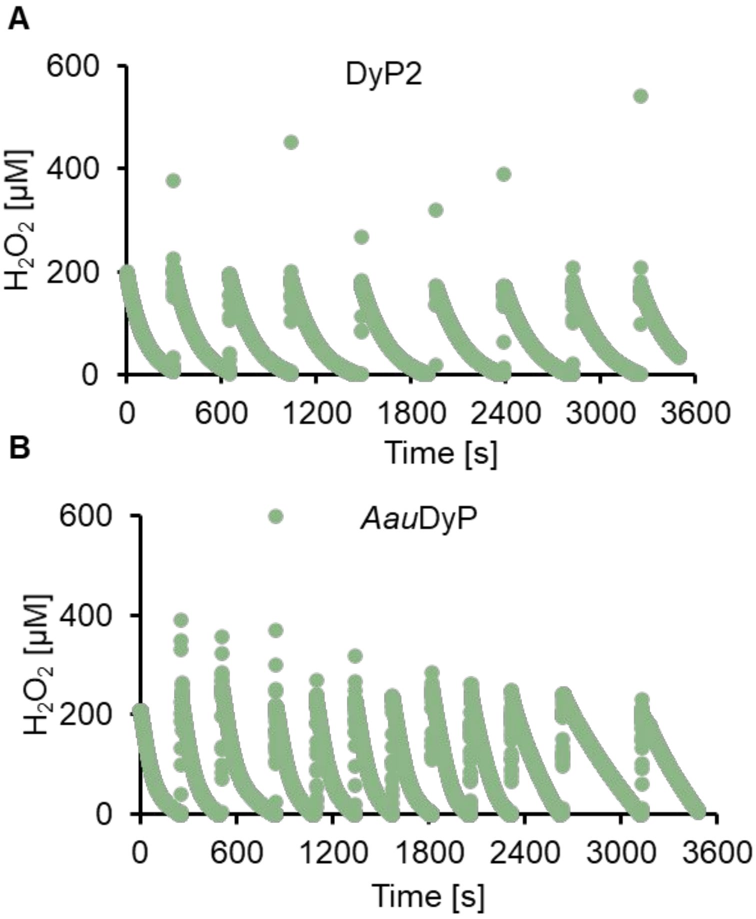

Figure S8: The continuous conversion of H_2O_2 by bacterial and fungal dye-decolourising peroxidases on untreated spruce. For each measurement the sensor was first calibrated by stepwise titration of 40 µM H_2O_2 to a final concentration of 200 µM while rotating at 2000 rpm in the 100 g L^{-1} substrate mixture at pH 4.5. The reaction was initiated by the addition of 0.1 µM of the enzyme. After complete conversion of H_2O_2, 200 µM of fresh H_2O_2 was repeatedly added to the reaction mixture for one hour. The H_2O_2 conversion of **A)** bacterial DyP2 and **B)** fungal *Aau*DyP on untreated spruce.

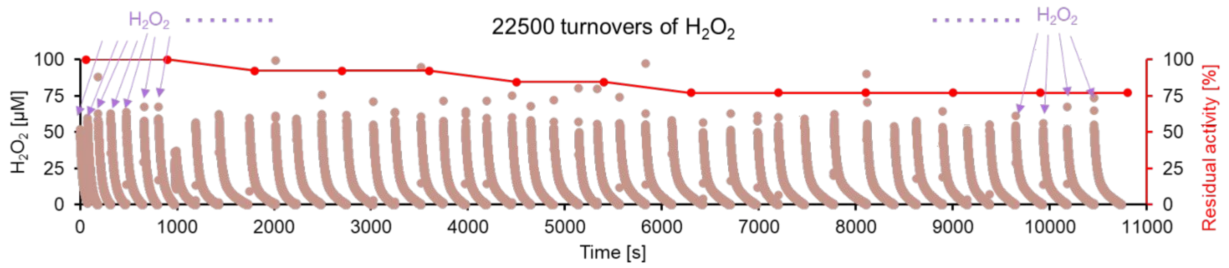

Figure S9: The continuous addition and conversion of 50 µM H_2O_2 by bacterial DyP2 on kraft lignin. The sensor was first calibrated by stepwise titration of 25 µM H_2O_2 to a final concentration of 50 µM while rotating at 2000 rpm in the 100 g L^{-1} kraft lignin mixture at pH 4.5. The reaction was initiated by the addition of 0.1 µM of the enzyme. After complete conversion of H_2O_2, 50 µM of fresh H_2O_2 was repeatedly titrated to the reaction mixture. After three hours, DyP2 performed 22500 turnovers of H_2O_2 while remaining 77% active.

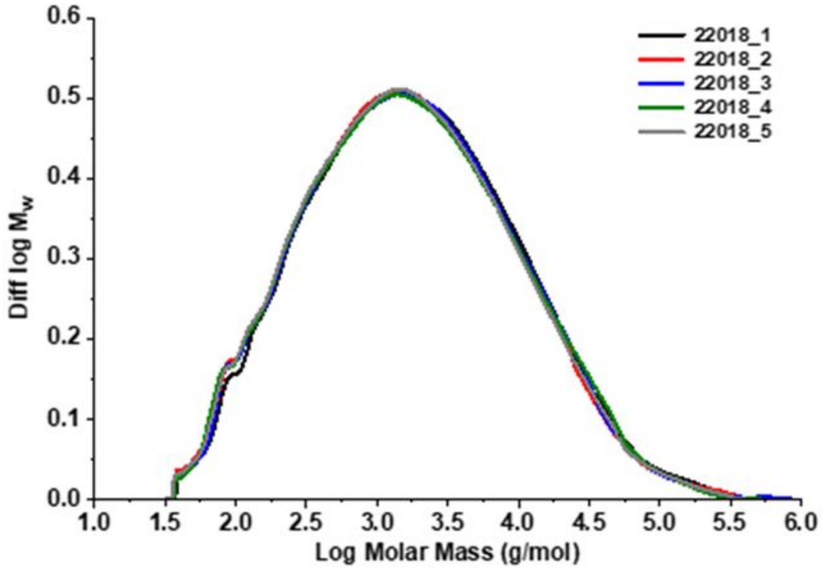

Figure S10: GPC-MALS of insoluble kraft lignin after one-hour enzymatic reaction. **1**- kraft lignin 100 g L^{-1}, **2**- kraft lignin 100 g L^{-1} with 0.1 µM of DyP2, **3**- kraft lignin 100 g L^{-1} with 200 µM of H_2O_2, **4**- kraft lignin 100 g L^{-1} with 0.1 µM of DyP2 and 200 µM of H_2O_2 after 200 s, **5**- kraft lignin 100 g L^{-1} with 0.1 µM of DyP2 and 200 µM of H_2O_2 after one hour. There was no significant difference between the enzymatically treated samples and the control samples.

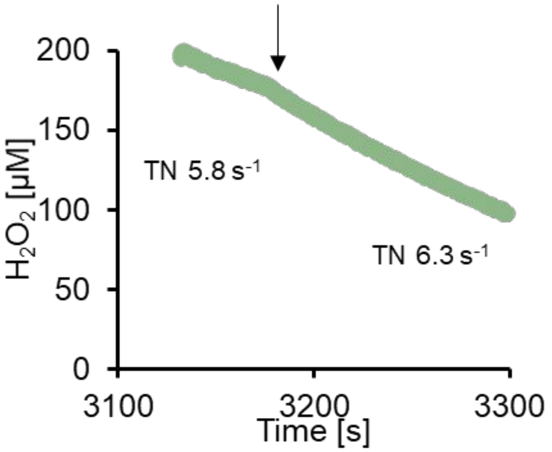

Figure S11: Fungal *Aau*DyP inactivation by substrate depletion. A fresh amount of *Aau*DyP indicated with a black arrow was added to the reaction mixture towards the end of the spruce reaction. Prior to the enzyme addition, the turnover number was 5.8 s^{-1}, but afterwards it stayed nearly identical (6.3 s^{-1}), suggesting that the reaction mixture did not contain suitable substrates for further enzymatic activity.

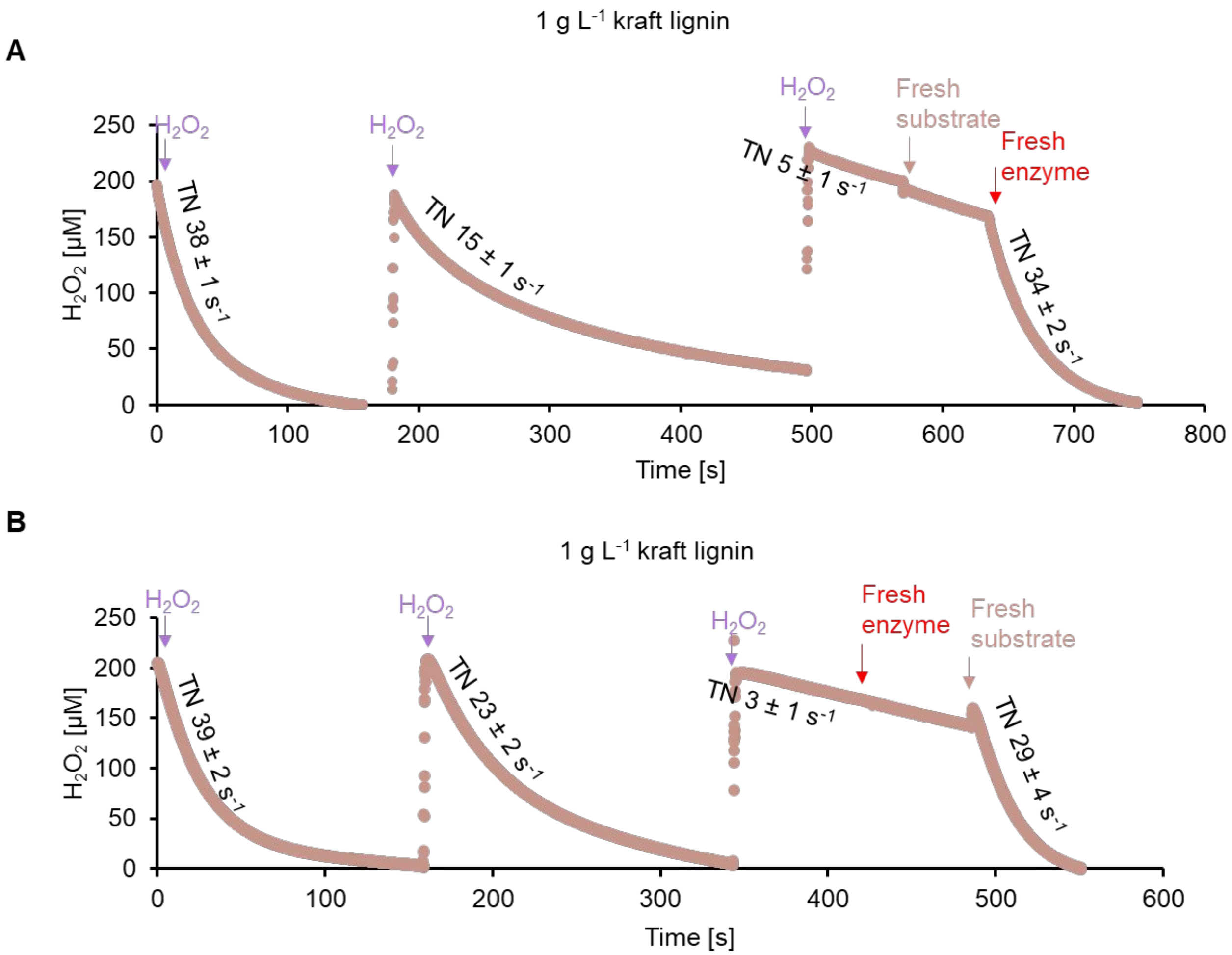

Figure S12: Bacterial DyP2 inactivation on kraft lignin. Enzymatic reactions were conducted with 1 g L^{-1} kraft lignin to achieve fast substrate consumption, but still performed the reaction under the saturated conditions. For each measurement the sensor was first calibrated by the stepwise titration of 40 µM H_2O_2 to a final concentration of 200 µM while rotating at 2000 rpm in the 1 g L^{-1} substrate suspension at pH 4.5. The reaction was initiated by the addition of 0.1 µM of the enzyme. After complete conversion of H_2O_2, 200 µM of fresh H_2O_2 was repeatedly added (indicated with the purple arrows) to the reaction mixture until a significant decrease in catalytic rates was observed. **A)** A fresh 100 µL of 100 g L^{-1} kraft lignin indicated with the brown arrow was added to the reaction mixture when decrease in catalytic rate was observed. The small "nick" in the graph indicates substrates addition, because of the small changes in the reaction volume. Prior to the substrate addition, the turnover number was 5 ±1 s^{-1}, but remained the same afterwards, suggesting that the enzyme was not active, despite adding fresh kraft lignin. When fresh 0.1 µM of DyP2 (red arrow) was added to the reaction mixture, H_2O_2 was rapidly converted, and the catalytic rate returned to its initial value. **B)** After the significant decrease in the catalytic rate, fresh 0.1 µM of DyP2 was added to the reaction mixture, but no increase in the turnover number was observed, indicating substrate depletion. Only when fresh 100 µL of 100 g L^{-1} kraft lignin (brown arrow) was added, the turnover number reached 29 ±4 s^{-1} which was comparable to the initial values before inactivation.

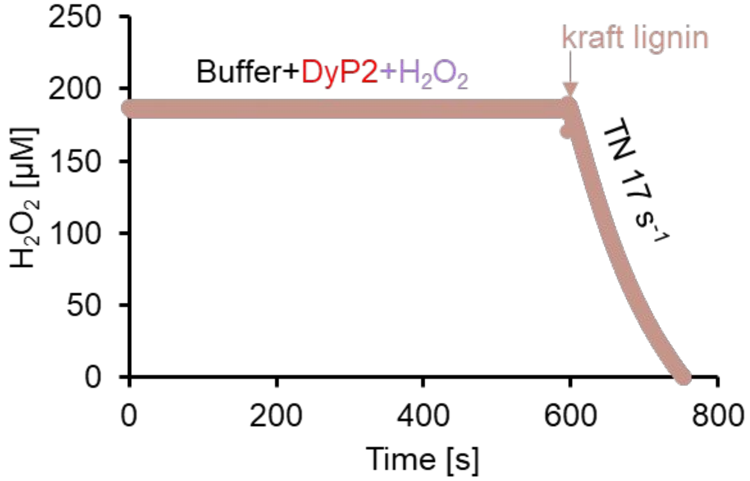

Figure S13: The effect of H_2O_2 on bacterial DyP2. The sensor was first calibrated by the stepwise addition of 40 μM H_2O_2 to a final concentration of 200 μM while rotating at 2000 rpm in the buffer at pH 4.5. After calibration, 0.1 μM of DyP2 was added and incubated with buffer and H_2O_2 for 10 minutes while rotating. Finally, 100 μL of 100 g L^{-1} kraft lignin (brown arrow) was added and catalytic rate was determined. The observed turnover number was 17 s^{-1}, which was almost half of that obtained without pre-incubation with H_2O_2, indicating that the presence of H_2O_2 in the absence of substrates causes the enzyme to lose activity.

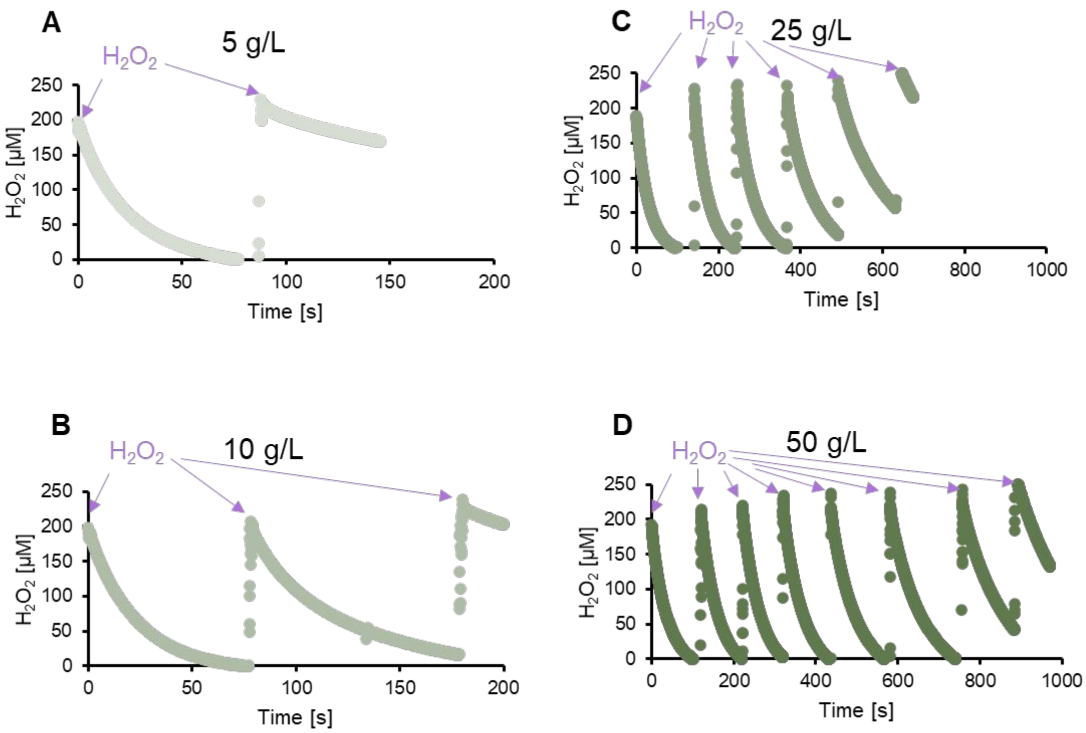

Figure

Figure S14: Bacterial DyP2 inactivation on untreated spruce. Enzymatic reactions were conducted with **A)** 5 g L^{-1}, **B)** 10 g L^{-1}, **C)** 25 g L^{-1}, **D)** 50 g L^{-1} of spruce. For each measurement the sensor was first calibrated by the stepwise addition of 40 µM H_2O_2 to a final concentration of 200 µM while rotating at 2000 rpm in the substrate mixture at pH 4.5. The reaction was initiated by the addition of 0.1 µM of the enzyme. After complete conversion of H_2O_2, 200 µM of fresh H_2O_2 (purple arrows) was repeatedly added to the reaction mixture until the notable decrease in the catalytic rates was observed. Due to the consistency of 100 g L^{-1} spruce, it was not possible to add small amounts of fresh substrate as performed with kraft lignin in Supplementary Figure 12. However, each panel shows that the more substrate is available, the slower the inactivation of the enzyme.

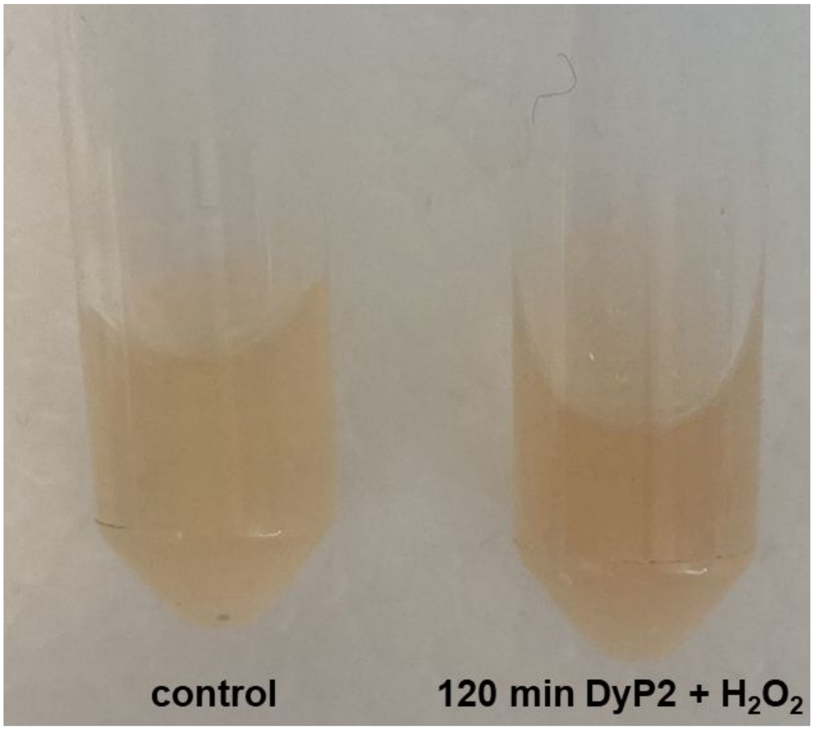

Figure S15: Visualization of soluble kraft lignin samples after two hours. The enzymatically treated samples obtained darker brown color after two hours compared to the kraft lignin control.

Table S1: Pre-processing parameters in MS DIAL.

MS-DIAL ver. 4.9.221218	
#Project	
MS1 Data type	Centroid
MS2 Data type	Centroid
Ion mode	Negative
Target	Metablomics
Mode	ddMSMS
#Data collection parameters	
Retention time begin	0
Retention time end	30
Mass range begin	0
Mass range end	1700
MS2 mass range begin	0
MS2 mass range end	1700
#Centroid parameters	
MS1 tolerance	0.01
MS2 tolerance	0.025
#Isotope recognition	
Maximum charged number	2
#Data processing	
Number of threads	5
#Peak detection parameters	
Smoothing method	Linear Weighted Moving Average
Smoothing level	8
Minimum peak width	8
Minimum peak height	2000
#Peak spotting parameters	
Mass slice width	0.1

Exclusion mass list (mass & tolerance)	
#Deconvolution parameters	
Sigma window value	0.5
MS2Dec amplitude cut off	0
Exclude after precursor	True
Keep isotope until	0.5
Keep original precursor isotopes	False
#MSP file and MS/MS identification setting	
MSP file	
Retention time tolerance	100
Accurate mass tolerance (MS1)	0.01
Accurate mass tolerance (MS2)	0.05
Identification score cut off	80
Using retention time for scoring	False
Using retention time for filtering	False
#Text file and post identification (retention time and accurate mass based) setting	
Text file	
Retention time tolerance	0.1
Accurate mass tolerance	0.01
Identification score cut off	85
#Advanced setting for identification	
Relative abundance cut off	0
Top candidate report	False
#Adduct ion setting	
[M-H]-	
[M+Na-2H]-	
[M+Cl]-	
[M+FA-H]-	
[M-CO2-H]-	
#Alignment parameters setting	
Retention time tolerance	1.5
MS1 tolerance	0.015
Retention time factor	0.5
MS1 factor	0.5
Peak count filter	0
N% detected in at least one group	50
Remove feature based on peak height fold-change	True
Sample max / blank average	5
Sample average / blank average	5
Keep identified and annotated metabolites	True
Keep removable features and assign the tag for checking	True
Gap filling by compulsion	True
#Tracking of isotope labels	
Tracking of isotopic labels	FALSE
#Ion mobility	
Ion mobility data	FALSE
